# Enhancement of broad-spectrum disease resistance in wheat through key genes involved in systemic acquired resistance

**DOI:** 10.3389/fpls.2024.1355178

**Published:** 2024-02-23

**Authors:** Shuqing Zhao, Mengyu Li, Xiaopeng Ren, Chuyuan Wang, Xinbo Sun, Manli Sun, Xiumei Yu, Xiaodong Wang

**Affiliations:** ^1^ State Key Laboratory of North China Crop Improvement and Regulation, College of Plant Protection, Hebei Agricultural University, Baoding, Hebei, China; ^2^ College of Agronomy, Hebei Agricultural University, Baoding, Hebei, China; ^3^ College of Life Sciences, Hebei Agricultural University, Baoding, Hebei, China

**Keywords:** wheat, systemic acquired resistance, genetic improvement, NPR1, *PR* genes

## Abstract

Systemic acquired resistance (SAR) is an inducible disease resistance phenomenon in plant species, providing plants with broad-spectrum resistance to secondary pathogen infections beyond the initial infection site. In *Arabidopsis*, SAR can be triggered by direct pathogen infection or treatment with the phytohormone salicylic acid (SA), as well as its analogues 2,6-dichloroisonicotinic acid (INA) and benzothiadiazole (BTH). The SA receptor non-expressor of pathogenesis-related protein gene 1 (NPR1) protein serves as a key regulator in controlling SAR signaling transduction. Similarly, in common wheat (*Triticum aestivum*), pathogen infection or treatment with the SA analogue BTH can induce broad-spectrum resistance to powdery mildew, leaf rust, *Fusarium* head blight, and other diseases. However, unlike SAR in the model plant *Arabidopsis* or rice, SAR-like responses in wheat exhibit unique features and regulatory pathways. The acquired resistance (AR) induced by the model pathogen *Pseudomonas syringae* pv. *tomato* strain DC3000 is regulated by *NPR1*, but its effects are limited to the adjacent region of the same leaf and not systemic. On the other hand, the systemic immunity (SI) triggered by *Xanthomonas translucens* pv. *cerealis* (*Xtc*) or *Pseudomonas syringae* pv. *japonica* (*Psj*) is not controlled by *NPR1* or SA, but rather closely associated with jasmonate (JA), abscisic acid (ABA), and several transcription factors. Furthermore, the BTH-induced resistance (BIR) partially depends on *NPR1* activation, leading to a broader and stronger plant defense response. This paper provides a systematic review of the research progress on SAR in wheat, emphasizes the key regulatory role of NPR1 in wheat SAR, and summarizes the potential of pathogenesis-related protein (*PR*) genes in genetically modifying wheat to enhance broad-spectrum disease resistance. This review lays an important foundation for further analyzing the molecular mechanism of SAR and genetically improving broad-spectrum disease resistance in wheat.

## Introduction

As early as 1901, researchers found that plants infected with pathogens develop higher levels of resistance to secondary infections (Görlach et al., 1996). Over the next 30 years, several descriptive studies were conducted, which collectively indicated the presence of systemic immunity in plants. Among them, the concept of SAR was widely accepted, referring to the ability of plants to develop broad-spectrum resistance against secondary pathogens during the response to primary pathogen infection (Görlach et al., 1996). Tobacco mosaic virus and its *Solanaceae* hosts were used in the early study of SAR (Görlach et al., 1996). With the deepening of research, SAR has been demonstrated to exist widely in various plants, and effectively combat viral, bacterial, and fungal diseases, and induce disease resistance responses with long-term and systematic nature (Zhang et al., 1999).

## SAR in *Arabidopsis* and rice

Usually caused by local infection of pathogenic bacteria, SAR is an important part of plant disease resistance system, and is an inducible broad-spectrum immunity of plant resistance to pathogenic bacteria, lacks specificity toward the initial infection (Görlach et al., 1996). When a non-lethal pathogen causes localized programmed cell death, it triggers the accumulation of the plant hormone SA and the expression of *PR* genes, thereby protecting the rest of the plant from secondary infections for weeks to months. SAR can even be transmitted to offspring through epigenetic regulation (Fu and Dong, 2013).

In the model plant *Arabidopsis thaliana*, infection with pathogenic microorganisms or treatment with SA and its analogs INA and BTH can induce SAR response, associated with the transcriptional activation of *PR* genes (Fu and Dong, 2013). Specifically, the SA receptor protein NPR1 in *Arabidopsis* is a key transcriptional regulatory factor for SAR. Upon infection with a primary pathogen or treatment with SA analogs, the intracellular SA level significantly increases, and NPR1 protein undergoes redox modification to translocate from cytoplasm to nucleus. After phosphorylation, it forms a complex with TGA transcription factors, promoting the expression of various *PR* genes and enhancing the plant resistance to secondary pathogens (Delaney et al., 1995; Mou et al., 2003). The endogenous level of SA in plant defense responses is largely dependent on the intensity of the hypersensitive response (HR) induced by the pathogen (Mou et al., 2003).

In *Arabidopsis*, the homologous proteins of NPR1, NPR3 and NPR4, participate in E3 ligase-mediated degradation of NPR1 in an SA-dependent manner (Fu et al., 2012). When the SA level is low, proteases bind to NPR4 and degrade NPR1. However, during pathogen infection, when the SA level increases, it competitively binds to NPR4. This binding leads to the accumulation of NPR1 and activates the NPR1-mediated defense response. When plants develop HR as part of the defense response, SA levels reach very high levels in the plant, and in this scenario, SA binds to NPR3 to promote its interaction with NPR1, ultimately leading to the transformation of NPR1 (Fu et al., 2012; Moreau et al., 2012). Recently, the crystal structure of the NPR1 protein in *Arabidopsis* has been resolved, revealing its existence as a “bird-shaped” dimer. NPR1-induced defense gene reprogramming in response to various biotic and abiotic stresses may involve not only TGA homodimers but also heterologous transcription activators, repressors, and lipid metabolites (Kumar et al., 2022).

In rice, infection with *Pseudomonas syringae* or treatment with BTH can induce SAR-like response against the rice blast fungus and is associated with the transcriptional activation of *PR* genes (Smith and Métraux, 1991; Schweizer et al., 1999) However, compared to *Arabidopsis*, rice has relatively higher endogenous levels of SA, which do not increase upon pathogen infection (Silverman et al., 1995; Chen et al., 1997). The protein interaction between rice NPR1 homolog (rNH1) and TGA transcription factors is conserved (Chern et al., 2001). Overexpression of the *Arabidopsis AtNPR1* gene in rice significantly enhances the broad-spectrum resistance of plant to various pathogens, including *Xanthomonas oryzae* pv. *oryzae* (*Xoo*)*, Magnaporthe oryzae*, and *Cochliobolus miyabeanus* (Quilis et al., 2008; Xu et al., 2017). Further studies indicate that the overexpression of the *rNH1* gene in rice not only enhances resistance to *Xoo* but also increases sensitivity to light and BTH treatment (Chern et al., 2005; Xu et al., 2017). Additionally, the WRKY transcription factor *OsWRKY45* is a key regulator of the SA/BTH signaling pathway independent of *rNH1* in rice (Shimono et al., 2007; Nakayama et al., 2013).

## SAR-like responses in *Triticeae* crops of wheat and barley

**Figure 1:**
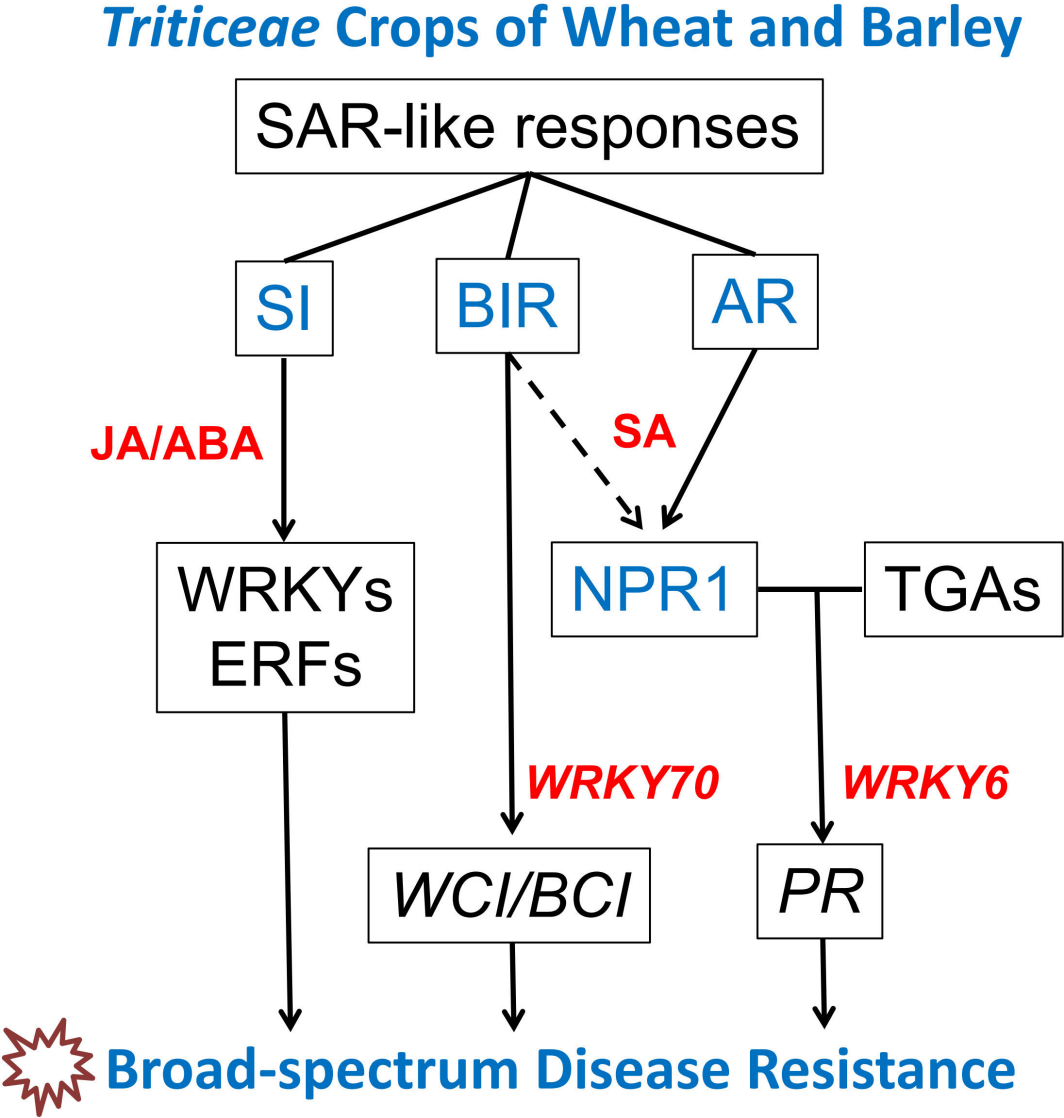
Regulatory network of SAR-like responses in *Triticeae* crops of wheat and barley. This network encompasses three SAR-like responses: acquired resistance (AR), systemic immunity (SI), and benzothiadiazole-induced resistance (BIR). Recent studies utilizing transcriptome sequencing and wheat transgenic lines have contributed to the prediction of this regulatory network. Understanding the molecular mechanisms that underlie SAR-like responses provides valuable gene resources for enhancing the broad-spectrum resistance of wheat to diseases such as stripe/leaf rust, powdery mildew, *Fusarium* head blight, *Fusarium* crown rot, sharp eyespot, and others.

In *Triticeae* crops of wheat and barley, both primary pathogen infection and BTH treatment can induce broad-spectrum resistance against diseases like powdery mildew, rust, and *Fusarium* head blight. However, SAR in *Triticeae* crops such as wheat and barley, when induced by pathogen infection or BTH treatment, displays distinct characteristics and regulatory pathways compared to model plants like *Arabidopsis* and rice. As a result, three different “SAR-like responses” have been observed in wheat and barley: acquired resistance (AR), systemic immunity (SI), and BTH-induced resistance (BIR) (Wang et al., 2018) (**Table 1**, **Figure 1**).

**Table 1:** Features and molecular mechanism of SAR response in *Arabidopsis*, rice, and *Triticeae* crops of wheat and barley.

SAR response	Inducible factor	Resistance features	Molecular mechanism	Reference
Arabidopsis	Infection of pathogen; Treatment of SA, INA, and BTH	HR induced by the pathogen, Enhanced resistance in systemic leaf, Increased SA content, Systemic.	Transcriptional activation of *PR* genes, Regulated by NPR1, Involvement of WRKY transcription factors.	Fu and Dong (2013)
Rice	Infection of *Pseudomonas syringae* pv *tomato* DC3000, Treatment of BTH	Pathogens induce HR, Plants exhibit high basal SA content, Systemic	*PR* gene transcription activation, WRKY45 independently regulates BTH-induced resistance	Smith and Métraux (1991); Schweizer et al. (1999); Shimono et al. (2007); Nakayama et al. (2013);
Wheat and barley acquired resistance (AR)	Infection of *Pseudomonas syringae* pv *tomato* DC3000,	Pathogenic bacteria trigger HR, Resistance is enhanced in adjacent areas of HR, SA content in necrotic leaves increases, Not systemic	*PR* gene transcription activation, Regulated by *NPR1,* Regulated by *WRKY6.*	Li et al. (2020); Li et al. (2022);
Barley systemic immunity (SI)	Infection of *Xanthomonas translucens* pv *cerealis* (*Xtc*) and *Pseudomonas syringae* pv *japonica* (*Psj*)	Pathogenic bacteria trigger HR, Resistance is enhanced in systemic leaf, Low association with SA, High association with JA and ABA, Systemic	Regulated independently of *NPR1,* Regulated by *WRKY* and *ERF*	Dey et al. (2014);
Wheat and barley BTH-induced resistance (BIR)	Treatment of BTH	Enhanced resistance to multiple fungal diseases, Systemic	Transcriptional activation of wheat *WCI* and barley *BCI* genes, Transcriptional activation of *PR* genes, Partial involvement of *NPR1,* Regulated by *WRKY70*	Görlach et al. (1996); Li et al. (2020); Hafez et al. (2014);

In *Triticeae* crops of wheat and barley, AR like the SAR in model plant can be observed in the neighboring area after injection with *Pseudomonas syringae* pv. *tomato* strain DC3000. This AR response enhances resistance against the secondary pathogen *M. oryzae* by upregulating *PR* genes (Colebrook et al., 2012). However, unlike SAR in *Arabidopsis*, the AR response in barley does not extend to other leaves, meaning it lacks systemic properties. Inducing AR response in wheat by injecting *P. syringae* DC3000 significantly enhances resistance to the highly virulent *Puccinia tritici* pathotype THTT (Gao et al., 2018). Overexpression of the wheat *NPR1* gene in barley (*wNPR1-OE*) significantly enhances resistance to the *M. oryzae* strain Guy11, while gene silencing of the barley *HvNPR1* gene (*HvNPR1-Kd*) significantly reduces AR response to Guy11, indicating that the *NPR1* gene directly regulates the AR response in barley. Using quantitative real-time polymerase chain reaction (qRT-PCR), it was found that the induction levels of barley *PR* genes, including *HvPR1b*, *HvPR2*, *HvPR3_Chit2b*, and *HvPR5*, were closely correlated with the expression levels of *NPR1* transgene in *wNPR1-OE* and *HvNPR1-Kd* barley transgenic plants (Wang et al., 2016). Further transcriptomic analysis revealed that the expression of *HvPR1*, *HvPR2*, *HvPR3*, *HvPR5*, *HvPR9*, and *HvPR13* genes was significantly induced in the AR response to DC3000 in barley, and the induction patterns were positively correlated with the expression levels of the *NPR1* gene, suggesting that these genes are regulated by the *NPR1* gene in the AR response (Gao et al., 2018).

By transcriptome sequencing, it was found that barley transcription factor gene *HvWRKY6* may act as a regulatory factor for AR in wheat (Li et al., 2020). The AR response was induced by injecting DC3000, and the resistance level of wheat transgenic lines overexpressing *HvWRKY6* (*HvWRKY6-OE*) to *M. oryzae* strain P131 was significantly improved. The expression levels of *TaPR1a, TaPR2* and *TaPR4b* genes in *HvWRKY6-OE* were significantly increased, indicating that *HvWRKY6* gene plays an important regulatory role in AR response. *HvWRKY6-OE* have shown enhanced resistance to *P. tritici* pathotype THTT, *Puccinia striiformis* f. sp. *tritici* pathotype CYR32, *Fusarium* crown rot, and sharp eyespot to varying degrees (Li et al., 2020; Li et al., 2022). Transcriptome sequencing analysis showed that *HvWRKY6* gene activated wheat defense response in a pathogen-independent manner. *HvWRKY6* partially activates the SAR-associated transcripts, including calcium-associated disease resistance pathways and part of the effector-triggered immunity (ETI) (Li et al., 2022). *HvWRKY6*-mediated resistance response is related to the activation of SA pathway and the inhibition of ABA and JA pathways (Li et al., 2022).

Injecting *Xanthomonas translucens* pv. *cerealis* (*Xtc*) or *Pseudomonas syringae* pv. *japonica* (*Psj*) into barley leaves can induce SI against *Xtc* infection in other systemic healthy leaves (Dey et al., 2014). However, unlike SAR in *Arabidopsis*, the SI process in barley is not induced by SA or BTH, but is closely associated with jasmonic acid (JA) and abscisic acid (ABA). Further studies have shown that the SI process in barley is regulated by several *WRKY* and *ERF* transcription factors but has a relatively low correlation with the *NPR1* gene.

BTH has been successfully developed as a commercial plant resistance inducer and applied in agricultural production. In *Arabidopsis* and tobacco, induction of resistance and *PR* gene expression by INA/BTH has been observed (Uknes et al., 1992; Lawton et al., 1995). It is likely that SA/INA/BTH induce SAR through the same signal transduction pathway (Görlach et al., 1996). Treatment of wheat with BTH induces systemic BIR against multiple diseases such as powdery mildew, leaf rust, and *Fusarium* head blight. However, most *PR* genes are insensitive to SA and BTH, while another class of BTH-induced genes, such as the wheat chemical induced (*WCI*) gene and the barley chemical induced (*BCI*) gene, may play important roles in the BIR (Beßer et al., 2000; Hafez et al., 2014). Transcriptome sequencing showed that the barley transcription factor gene *HvWRKY70* may act as a regulatory factor of BIR in wheat. BTH-induced SAR reaction showed that several *PR* genes and *BCI* genes in wheat transgenic lines overexpressing *HvWRKY70* (*HvWRKY70-OE*) were significantly up-regulated by BTH, indicating that *HvWRKY70* gene was involved in the regulation of BIR. In addition, *HvWRKY70-OE* showed increased resistance to *P. striiformis* f. sp. *tritici* pathotype CYR32 and *Blumeria graminis* f. sp. *tritici* pathotype E20 (Li et al., 2020).

## Interactors of the NPR1 protein in plant-pathogen interaction

NPR1, as a SA receptor protein, possesses three conserved domains: the N-terminal broad-complex, tramtrack, and brica-brac/poxvirus, zinc finger (BTB/POZ) domain, the central ankyrin-repeat (ANK) domain, and the C-terminal NPR1/NIM1-like domain. The BTB/POZ domain is a potential target for the E3 ubiquitin degradation pathway (Petroski and Deshaies, 2005), while the ANK domain is mainly involved in the interaction with TGA transcription factors (Cao et al., 1997; Sedgwick and Smerdon, 1999). The NPR1/NIM1-like domain participates in SA binding along with the BTB/POZ domain (Wu et al., 2012).

NIMIN is a protein family first discovered in *Arabidopsis* and plays a crucial role in regulating the NPR1-mediated immune signaling pathway. It finely regulates the dynamic defense process against diseases. Hermann et al. reported that during different stages of SAR, different NIMIN-NPR1 complexes are formed to promote the activation of defense genes. In normal plant growth, the binding of NIMIN3 to NPR1 prevents excessive activation of immune responses (Hermann et al., 2013). However, upon pathogen attack, NIMIN2 and NIMIN1 sequentially bind to NPR1, promoting the binding with SA and the expression of *PR* genes, thereby enhancing plant immune responses (Maier et al., 2011). NIMIN proteins regulate the dynamic balance of NPR1 and contribute to the rapid upregulation of defense genes, ensuring successful resistance against invading pathogens (Hermann et al., 2013). TEOSINTE BRANCHED 1, CYCLOIDEA, PCF (TCP) transcription factors are also essential participants in the SA signaling pathway. NPR1 interacts with nuclear TCP transcription factors TCP8, TCP14 and TCP15, promoting SAR function. TCP15 directly binds to the TCP binding site in the *PR5* promoter, enhancing *PR5* expression, contributing to ETI, and playing an important role in SAR (Li et al., 2018). NPR1 has also been reported to interact with cyclin-dependent kinase 8 (CDK8) and WRKY18 in *Arabidopsis*, and SA can promote their interaction. CDK8 facilitates the expression of *NPR1* and *PR1* genes by recruiting RNA polymerase II to their promoter and coding regions. NPR1, in turn, recruits CDK8, promoting its own and target gene expression and contributing to the establishment of plant immunity (Chen et al., 2019).

Both fungal pathogen *Colletotrichum fructicola* effector protein CfEC12 and apple MdNPR1 interact with NIM1-interacting (MdNIMIN2) protein. CfEC12 competes with MdNIMIN2 in binding to the 13-63 amino acid position of MdNPR1, thereby suppressing the expression of downstream *PR* genes and immune responses (Shang et al., 2023). NPR1 plays a key role in limiting co-infection of TuMV, a member of the largest RNA virus genus in plant, and this resistance is counteracted by viral RNA-dependent RNA polymerase nuclear inclusion body B (NIb). NIb interacts with NPR1 and targets its SUMO interacting motif 3 (SIM3); NIb interferes with NPR1-SIM3 interaction and subsequent SUMoization; NIb also affects the SUMO-dependent phosphorylation of NPR1; targeting NPR1-SIM3 is a conserved ability of NIBs from different descendant viruses. *Potyvirus* uses NIb to suppress NPR1-mediated resistance by disrupting NPR1 SUMO (Liu et al., 2023a). The RXLR effector protein RXLR48 in *Phytophthora capsica* interacts with NPR1 and inhibits plant defense. RxLR48 promotes nuclear localization of NPR1 and inhibits its proteasome-mediated degradation, suggesting that RxLR48 inhibits SA signaling by targeting the transcriptional regulator NPR1 (Li et al., 2019). Using yeast two-hybrid screening, a virulent effector protein, PNPi, that directly targets the NPR1 protein was identified in wheat rust, inhibiting the interaction between NPR1 and TGA2, and suppressing wheat SAR (Wang et al., 2016). There were 19 PNPi-like secreted proteins with RlpA-like double-psi beta-barrel (DPBB_1) conserved structure in wheat leaf rust. Twelve PNPi-like effector protein genes were cloned and their interaction with wheat NPR1 protein was verified by yeast two-hybrid system. Among them, four PNPi-like effector proteins could interact with wheat NPR1 protein (Zhao et al., 2020).

## NPR1 protein in *Triticeae* crops of wheat and barley

In the *Triticeae* crops of wheat and barley, significant progress has been made in the study of *NPR1* gene (**Table 2**). For example, overexpression of *Arabidopsis AtNPR1* or oat *ScNPR1* in common wheat significantly enhances plant resistance against *Fusarium graminearum* (Gao et al., 2013; Yu et al., 2017). The interaction between NPR1 homologous proteins and TGA transcription factor homologous proteins is highly conserved in wheat and rice, suggesting that wheat NPR1 homologous proteins have conserved disease resistance functions (Cantu et al., 2013). Bioinformatics analysis revealed the presence of nine *NPR1* homologous genes (*TaNPR1*) in wheat, with six members located on the homoeologous group 3 chromosomes named *TaG3NPR1*, and three members located on the homoeologous group 7 chromosomes named *TaG7NPR1*. *TaG3NPR* regulates the expression of *PR* genes in the SA signaling pathway. Additionally, a novel fusion pattern of NPR1 protein, as NPR1 fused with nucleotide-binding adaptor shared by APAF-1, R proteins, and CED-4 (NB-ARC-NPR1) was discovered on the 7A chromosome in common wheat (Ta7ANPR1). Under biotic stress conditions, the *Ta7ANPR1* gene simultaneously transcribes two mRNAs, one encoding an NB-ARC protein and the other encoding an NB-ARC-NPR1 fusion protein. The *Ta7ANPR1* gene negatively regulates plant defense responses against wheat rust through the NB-ARC-NPR1 fusion protein (Wang et al., 2020b). Calcineurin B-like interacting protein kinases (CIPKs) have been shown to be essential for biological stress tolerance in plant-pathogen interactions. A CIPK homolog, *TaCIPK10*, was identified and cloned from wheat. TaCIPK10 physically interacts and phosphorylates with AtNPR3/4 homologous TaNH2 to regulate wheat resistance to *P. striiformis* f. sp. *tritici* (Liu et al., 2019a).

**Table 2:** Functional characterized *NPR* genes in wheat.

Gene Name	Gene accession	Role in wheat resistance	Reference
*NPR1-3A/3B/3D*	TraesCS3A02G105400 TraesCS3B02G123800 TraesCS3D02G107500	Positive regulator of AR and BIR, Targeted by rust effector PNPi	Wang et al. (2016); Liu et al. (2019b);
*TaG3NPR1*	KAF7021350 KAF7028198	Regulation of expression of *PR* genes in SA signaling pathway	Wang., et al. (2020b);
*TaG7NPR1/NB-ARC-NPR1*	KAF7046550 KAF7105730 TraesCS7D02G023000	Simultaneously transcribes two mRNAs and negative regulation of plant defense response to wheat rust disease	Wang., et al. (2020b);
*TaNH2*	KU736862	Interacted with TaCIPK10 and enhance disease resistance to wheat stripe rust	Liu et al. (2019a);

Furthermore, it has been reported that about 40 *NPR1* homologous genes or *NPR* family coding genes can be identified in *Triticum aestivum*, *Triticum urartu*, *Triticum dicoccoides*, and *Aegilops tauschii*. *NPR1* homologous genes exhibit good collinearity in common wheat and its close relatives. Based on RNA-seq data, *TaNPR1* homologous genes exhibit different tissue-specific expression patterns, and *TaNPR1-A/B/D*, *TaNPR3-A/B/D*, and *TaNPR4-A/B/D* are significantly induced under biotic stress conditions (Liu et al., 2019b). Through gene expression profile analysis, three *NPR1* homologous genes named *TaNPR1*, *TaNPR2*, and *TaNPR3* were cloned from wheat near-isogenic lines resistant to *Fusarium* head blight (Yang et al., 2013). Among them, *TaNPR1* and *TaNPR3* showed significant upregulation in response to *Fusarium graminearum*, suggesting their involvement in wheat defense against *Fusarium* head blight. Association analysis using a natural population consisting of 178 winter wheat genotypes revealed that two *NPR* genes located on the 2AL and 2DL chromosomes of common wheat were associated with resistance to *Fusarium* head blight (Diethelm et al., 2014). Both SA and the biocontrol agent *Trichoderma* induce the expression of the wheat *NPR1* gene and enhance plant resistance against powdery mildew (Ahangar et al., 2017).

## *PR* genes act as the downstream of SAR

PR proteins are a class of water-soluble proteins produced by plants in response to pathogen invasion or non-biological stimuli. When subjected to biotic or abiotic stress, the expression levels of *PR* genes rapidly increase and are often used as markers of plant immune activation. Numerous studies have shown that PR proteins play important roles in plant disease resistance and SAR (Hamamouch et al., 2011). To date, a total of 18 *PR* gene families have been identified in various plant species in response to different pathogens (van Loon et al., 2006; Ferreira et al., 2007; Sels et al., 2008; Wang et al., 2018). In addition, *PR* gene has been widely used as indicator gene for wheat resistance response to monitor the intensity of resistance response (Wu et al., 2019).

The PR1 protein family, as the earliest identified defense-related proteins, has been extensively studied for its disease resistance mechanisms. *PR1* homologous genes (*PR1a* and *PR1b*) have been successfully cloned in wheat and barley and induced to express by a variety of pathogens (Molina et al., 1999; Gao et al., 2015). These genes are considered as key downstream regulatory genes of NPR1 in SAR-like responses in *Triticeae* crops of wheat and barley (Wang et al., 2016). RNA-seq transcriptome sequencing was performed on resistant wheat line carrying leaf rust resistance gene *TcLr19* and susceptible wheat variety “Chinese Spring” inoculated with the *P. tritici* pathotype PHNT, and seven SA-induced *TaPR1* genes associated with plant disease resistance were identified. qRT-PCR results showed that among these *TaPR1* genes, *TaPR1-4* had the largest induction effect by the infection of leaf rust (Wang et al., 2022a). The *TdPR1.2* gene identified from *Trticum turgidum* also plays a vital role in enhancing plant resistance to abiotic stress (Ghorbel et al., 2021). The expression and genetic polymorphisms of *PR1, PR2, PR4, PR9* and *PR10* in sixteen Egyptian wheat genotypes were analyzed to clarify the expression mechanism of *PR* genes during stripe rust infection (Esmail et al., 2020).

The PR1 protein plays a pivotal role as an interaction hub in the extracellular space. TaPR1a protein in wheat interacts with lipid transfer protein TaLTP3 (PR14) in the extracellular matrix, and overexpression of *TaLTP3* in wheat transgenic line can specifically activate the transcription of *TaPR1a* gene, as well as multiple plant hormone pathways including SA, JA, and auxin, providing new insights into the synergistic mechanism of PR proteins (Zhao et al., 2021). TaPR1-4 interacts with thaumatin-like protein TaPR5/TaTLP1 through the αIV helix and participates in the defense process against wheat leaf rust through the CAPE1 motif (Wang et al., 2022a).

Research has shown that wheat TaPR1 protein can directly interact with the fungal toxin ToxA produced by the tan spot pathogen (*Pyrenophora tritici-repentis*) and mediate the induction of necrotic reactions in susceptible wheat (Lu et al., 2014). The effector SnTox3 from *Parastagonospora nodorum* elicits a strong necrotic response in susceptible wheat and also interacts with wheat TaPR-1, SnTox3 prevented CAPE1 from being released from TaPR1 *in vitro*, SnTox3 independently induced necrosis through Snn3 recognition, and inhibited host defense through direct interaction with the TaPR1 protein (Sung et al., 2021). The wheat transgenic lines overexpressing *TaPR1a* gene (*TaPR1a-OE*) showed increased resistance to both wheat leaf rust and stripe rust. By targeting TaPR1a protein in the extracellular space, wheat rust effector protein PNPi inhibits plant disease resistance and is conducive to rust infection (Bi et al., 2020).

Significant efforts have been dedicated to the genome-wide identification of *PR1* genes in wheat. Twelve *PR-1* genes encoding the CAP superfamily domain were identified in the genome of the *Triticum turgidum* subspecies. Phylogenetic analysis showed that *PR1* gene could be divided into three groups according to variations in conserved domain. Most TdPR1 proteins present an N-terminal signaling peptide. The expression profile analysis showed that the *PR-1* gene family was organism specific and could be induced by different abiotic stresses (Zribi et al., 2023). Bioinformatics tools and RNA sequencing discovered 86 potential wheat *TaPR1* genes, and *TaPR1* genes were involved in SA signaling pathway, MAPK signaling pathway, and phenylalanine metabolism in response to infection of *P. striiformis* f. sp. *tritici* pathotype CYR34. One particular gene, *TaPR1-7*, was found to be associated with resistance to *P. striiformis* f. sp. *tritici* in a biparental wheat population (Liu et al., 2023b). *TaPR1*, as the representative of wheat SAR downstream defense related protein genes, holds significant potential and is deserving of further exploration and investigation.

*PR2* and *PR3* genes encode β-1,3-endoglucanases and chitinase proteins, respectively. Molecular docking analysis of β-1,3-endoglucanases and chitinase proteins revealed key amino acid residues involved in ligand binding and important interactions, which may play an important role in plant defense against fungal pathogens (Numan et al., 2021). The PR4 family features a Barwin domain at the C-terminus, which endows the host plant with disease resistance. A total of four *PR-4* genes were identified from the genome of the Qingke (*Hordeum vulgare* L. var. *nudum*) by HMM analysis. Expression profile analysis confirmed that *PR-4* was involved in the defense response to drought, cold, and powdery mildew infection, and the transcription of two barley *PR4* genes were differentially regulated by MeJA and SA (Wang et al., 2022b).

Wheat thaumatin-like protein (TaTLP/TaPR5) are secreted into the apoplastic space, and when stimulated by biological or abiotic stresses, their expression levels increase rapidly, and they show antifungal activity in various plant species, which is an important component of plant SAR and a sign of plant disease resistance (Han et al., 2023). Wheat TaTLP1 is involved in the resistance to leaf rust. TaPR1 and TaTLP1 also have direct protein interaction in the extracellular space, positively regulating wheat resistance to leaf rust in a reactive oxygen species (ROS)-dependent and direct germicidal manner (Wang et al., 2022a). Leaf rust effector protein Pt_21 directly targets wheat TaTLP1 and inhibits host defense response by inhibiting the antifungal activity of TaTLP1 (Wang et al., 2023).

Currently, other *PR* genes in barley and wheat have been rarely reported. However, considering the research potential and application prospects of *PR* genes in other crops, they hold great promise for genetic improvement of wheat disease resistance. Drawing from information on *PR* genes reported in other plant species, we conducted a preliminary prediction and classification of 18 *PR* gene families in wheat (**Table 3**).

**Table 3:** Predicted and characterized *PR* genes in wheat in this study.

Gene Name	Encoded protein	Gene accession	Role in wheat resistance	Reference
*PR1*	Secretion protein with C-terminal CAPE1 peptide	TraesCS5A02G183300, TraesCS7D02G201400, TraesCS7B02G105300, TraesCS7A02G198900, TraesCS7A02G198800, TraesCS7D02G201300, TraesCS7B02G105200, TraesCS7B02G110000, TraesCS5D02G259800, TraesCS7D02G161200	Interact with PR5, Interact with LTP3 (PR14), Targeted by rust effector PNPi, Positive regulator in resistance to leaf rust and stripe rust, Downstream of AR, Downstream of BIR.	Zhao et al. (2021); Wang., et al. (2020a); Bi et al. (2020); Sung et al. (2021)
*PR2*	β-1,3-endoglucanases	TraesCS3D02G478300, TraesCS3D02G478000, TraesCS3B02G529700, TraesCS2D02G349400, TraesCS3A02G483000, TraesCS3B02G529300, TraesCS7B02G105100, TraesCS7B02G105000, TraesCS7B02G104900	*Pst-milR1* targets silencing wheat TaPR2, Downstream of AR, Downstream of BIR.	Numan et al. (2021);
*PR3/PR8/PR11*	Chitinase	TraesCS2D02G349400, TraesCS2D02G349000, TraesCS2D02G348900, TraesCS2B02G369100, TraesCS2A02G350800, TraesCS3D02G260500, TraesCS3D02G260300, TraesCS3B02G293200, TraesCS3A02G260200, TraesCS2A02G350900, TraesCS3B02G293400, TraesCS3A02G260100	Downstream of AR, Downstream of BIR.	van Loon et al. (2006); Giżyńska et al. (2018); Numan et al. (2021);
*PR4*	Chitinase and chitin-binding proteins	TraesCS3D02G524700, TraesCS3B02G584700, TraesCS3A02G517100	Inhibite growth of *Fusarium culmorum,* Downstream of AR	Wang et al. (2022b);
*PR5*	Thaumatin-like protein	TraesCS7B02G417700, TraesCS5B02G016000, TraesCS5B02G015700, TraesCS5B02G015500, TraesCS5A02G017900, TraesCS7D02G551400, TraesCS7B02G483400, TraesCS7A02G558500, TraesCS5A02G018200, TraesCS5A02G019100, TraesCS5A02G019000, TraesCS5A02G018900, TraesCS5A02G018800, TraesCS5A02G018700, TraesCS5A02G018600, TraesCSU02G146600, TraesCS6B02G473800, TraesCS4A02G498000, TraesCS2A02G110300	Interact with PR1, Targeted by leaf rust effector Pt_21, Positive regulator in resistance to leaf rust, Downstream of AR, Downstream of BIR.	Wang., et al. (2020a); Wang., et al.(2022a); Wang., et al. (2023)
*PR6*	Protease inhibitor	TraesCS1A02G265600, TraesCS1D02G266000, TraesCS1B02G276800, TraesCS1B02G276300, TraesCS1B02G276200, TraesCS1A02G265800	Unknown	Gaddour et al. (2001); Gao et al. (2013);
*PR7*	Endogenous protease	TraesCS5A02G520700, TraesCS5A02G693100, TraesCS4D02G456100, TraesCS4D02G456000, TraesCS4B02G352100,	Unknown	Jordá et al. (2000);
*PR9*	Peroxidase	TraesCS2B02G124800, TraesCS2D02G107800, TraesCS2B02G125200, TraesCS2A02G107500	Induce by powdery mildew, Downstream of AR.	Passardi et al. (2004);
*PR10*	Ribonuclease-like protein	TraesCS5D02G102700, TraesCS5B02G096300, TraesCS5A02G090600	Unknown	Aglas et al. (2020);
*PR12*	Small cysteine-rich antifungal protein	TraesCS2D02G047800, TraesCS2B02G062100, TraesCS2A02G048900	Unknown	Terras et al. (1995);
*PR13*	Thionins	TraesCS5B02G228600, TraesCS1A02G398200, TraesCS5A02G230000, TraesCS1D02G405700, TraesCS1B02G426100, TraesCS5A02G229900, TraesCS5A02G229800, TraesCS7D02G008100, TraesCS4A02G492000, TraesCS4A02G491800, TraesCSU02G200700, TraesCSU02G219800, TraesCSU02G066300, TraesCS4A02G491900, TraesCSU02G066400, TraesCS4A02G491700, TraesCSU02G219800, TraesCSU02G193300, TraesCS1B02G426000, TraesCS1D02G405600, TraesCS5D02G473800, TraesCS5B02G471300	Downstream of AR	Epple et al. (1995);
*PR14*	Lipid transfer protein	TraesCSU02G253500, TraesCSU02G056900, TraesCSU02G056700, TraesCS3B02G064000, TraesCS3B02G063700, TraesCS3B02G063500, TraesCS3B02G063100, TraesCSU02G258000, TraesCSU02G147300, TraesCS3B02G064300, TraesCS3B02G064200, TraesCS3B02G063900, TraesCSU02G056600, TraesCSU02G251500, TraesCSU02G237900, TraesCSU02G154200, TraesCSU02G147200, TraesCSU02G147100, TraesCS3B02G064100, TraesCS3B02G063600, TraesCS3B02G063400, TraesCS3B02G063200, TraesCS3B02G063000, TraesCS3B02G062700, TraesCS3B02G062600, TraesCS3B02G064400	PR14 (LTP3) activates *PR1* transcription, Downstream of BIR.	Blein et al. (2002); Bi et al. (2020); Zhao et al. (2021);
*PR15*	Oxalate oxidase	TraesCS4D02G032200, TraesCS4D02G032000, TraesCS4D02G031700, TraesCS4D02G031600, TraesCS4B02G033600, TraesCS4B02G033400, TraesCS4B02G033300, TraesCS4B02G033200, TraesCS4B02G033100, TraesCS4A02G181700, TraesCS4D02G030800, TraesCS4A02G279300, TraesCS4A02G279200, TraesCS4A02G279100, TraesCS3B02G282500, TraesCS3B02G282400	Unknown	Gregersen et al. (1997);
*PR16*	Oxalate oxidase-like protein	TraesCSU02G256400, TraesCSU02G253400, TraesCSU02G245800, TraesCSU02G238600, TraesCSU02G222000, TraesCSU02G172100, TraesCSU02G161300, TraesCSU02G152900, TraesCSU02G152800, TraesCSU02G152000, TraesCSU02G151939, TraesCSU02G151900, TraesCSU02G150800, TraesCSU02G150700, TraesCSU02G150600, TraesCSU02G145700, TraesCSU02G145600, TraesCSU02G145500, TraesCSU02G145300, TraesCSU02G128800, TraesCSU02G128700, TraesCSU02G128600, TraesCSU02G128500, TraesCSU02G128400, TraesCSU02G128300, TraesCSU02G128200, TraesCSU02G128100, TraesCSU02G128000, TraesCSU02G127900, TraesCS5A02G545400, TraesCS5A02G545300, TraesCS5A02G545250, TraesCS5A02G545200, TraesCS5A02G544521, TraesCS5A02G544500, TraesCS5A02G544400, TraesCS5A02G544200, TraesCS5A02G544196, TraesCS5A02G544189, TraesCS5A02G544100, TraesCS5A02G544000, TraesCS5A02G543800, TraesCS5A02G543756, TraesCS5A02G543700, TraesCS5A02G543600, TraesCS4B02G378400, TraesCS4B02G378300, TraesCS4B02G378200, TraesCS4B02G378100, TraesCS4B02G378000, TraesCS4B02G377900, TraesCS4B02G377800, TraesCS4B02G377700, TraesCS4B02G377500, TraesCS4B02G377400, TraesCS4B02G377300, TraesCS4B02G377200, TraesCS4B02G377100, TraesCS4B02G377000, TraesCS4B02G376900, TraesCS4B02G376700	Unknown	Zhou et al. (1998);
*PR17*	Plant basic secretory family protein	TraesCS6D02G072100, TraesCS1D02G174100, TraesCS6B02G105400, TraesCS1A02G166600, TraesCS6A02G078400, TraesCS1B02G183100	Downstream of BIR	Christensen et al. (2002); Zhang et al. (2012);
*PR18*	Carbohydrate oxidases FAD-binding Berberine family protein	TraesCS7B02G273700, TraesCS7D02G368800, TraesCS7A02G353900, TraesCS4D02G101000, TraesCS4B02G104000, TraesCS4A02G212100, TraesCS2A02G542600, TraesCS5A02G555000, TraesCS2B02G572400, TraesCS2D02G543700, TraesCSU02G034600, TraesCS4B02G391900, TraesCS4B02G358600, TraesCS7D02G393900, TraesCS7B02G300200, TraesCS7A02G400100, TraesCS7B02G273600, TraesCS7D02G368700, TraesCS7B02G273500, TraesCS3A02G066000LC, TraesCS7A02G354000, TraesCS7D02G368500, TraesCS7A02G126600, TraesCS2B02G145300, TraesCS3D02G113700, TraesCS7D02G124500, TraesCS5A02G261400, TraesCS3A02G111600, TraesCS7B02G273800, TraesCS2D02G126200, TraesCS7D02G369000, TraesCS5D02G268800, TraesCS7D02G472000, TraesCS3B02G132200, TraesCS2A02G052700, TraesCS2A02G123200, TraesCS7B02G388200, TraesCS7A02G484600, TraesCS7A02G353800, TraesCS7B02G273900, TraesCS2D02G052300, TraesCS2B02G066900, TraesCS2A02G052800, TraesCS2D02G126400, TraesCS2D02G052500, TraesCS2B02G145500, TraesCS2B02G066800, TraesCS2A02G052600, TraesCS2A02G052900, TraesCS2D02G052700	Unknown	Custers et al. (2004);

## Conclusion and future prospective

The SAR-like responses observed in *Triticeae* crops of wheat and barley (AR, SI, and BIR) exhibit significant differences compared to those in *Arabidopsis* and rice. In recent years, studies on the *NPR1* homologous genes in wheat have provided preliminary clues to understanding the molecular mechanisms underlying these differences. The key regulatory factors and downstream functional proteins in SAR, including the SA receptor proteins NPR3/4, WRKY transcription factors, and PR proteins, still require further exploration. Uncovering the key nodal genes involved in SAR-like responses in wheat, as well as the co-regulated downstream genes involved in these biological processes, will provide important genetic resources for broad-spectrum disease resistance improvement in wheat. Additionally, with the continuous advancement of genomics and the widespread application of gene editing technologies, knockout of key negative regulatory genes involved in SAR-like responses in wheat can generate innovative disease-resistant germplasm resources, demonstrating significant research prospects and application potential.

## Author contributions

SZ: Data curation, Investigation, Visualization, Writing – original draft, Software. ML: Data curation, Investigation, Software, Visualization, Writing – original draft. XR: Data curation, Investigation, Writing – original draft. CW: Data curation, Investigation, Writing – original draft. XS: Formal analysis, Funding acquisition, Supervision, Writing – review & editing. MS: Formal analysis, Funding acquisition, Supervision, Writing – review & editing. XY: Formal analysis, Supervision, Writing – review & editing. XW: Conceptualization, Funding acquisition, Project administration, Supervision, Writing – review & editing, Formal analysis.
